# Ligation of Innominate Artery

**Published:** 1868-02-01

**Authors:** Moses Gunn

**Affiliations:** Prof. Surgery Rush Med. Col.; Chicago


### LIGATION OF INNOMINATE ARTERY.—A SUGGESTION.

BY MOSES GUNN, M.D., PROF. SURGERY RUSH MED. COL.

THE full recovery of a patient, after ligation of the unnamed vessel, which occurred in New Orleans two or three years since has inspired a hope of the operation which for a long time past had ceased to exist as a general sentiment. But even in that successful case, the patient was in imminent danger from secondary hæmorrhage, which must ever menace the operation of *firm* ligature of the vessel.

It is not probable that the operation will be required for any thing, save the cure of aneurism ; and for this, *complete* arrest of the current of circulation is not necessary. I therefore suggest the application of a silver ligature to the vessel for the purpose of *constricting*, but not completely *occluding* it. The vessel should be constricted to about the size of the radial artery. By such application there would be no strangulation of the included portion of the walls ; and the metallic ligature would not be likely to excite ulceration. The ligature should be cut short and the wound closed accurately. By this procedure we should probably be able to obtain perfect healing of the wound before the usual period for secondary hæmorrhage. It is not probable, either, that that secondary hæmorrage would occur, even though the wound was not healed. I shall put the plan on trial should opportunity offer.

